# The clinical value of neutrophil-to-lymphocyte ratio (NLR), systemic immune-inflammation index (SII), platelet-to-lymphocyte ratio (PLR) and systemic inflammation response index (SIRI) for predicting the occurrence and severity of pneumonia in patients with intracerebral hemorrhage

**DOI:** 10.3389/fimmu.2023.1115031

**Published:** 2023-02-13

**Authors:** Rui-Hong Wang, Wan-Xin Wen, Ze-Ping Jiang, Zhen-Ping Du, Zhao-Hui Ma, Ai-Li Lu, Hui-Ping Li, Fang Yuan, Shi-Biao Wu, Jian-Wen Guo, Ye-Feng Cai, Yan Huang, Li-Xin Wang, Hong-Ji Lu

**Affiliations:** ^1^ The Second Clinical College of Guangzhou University of Chinese Medicine, Guangzhou, China; ^2^ Department of Neurocritical Care, The Second Affiliated Hospital of Guangzhou University of Chinese Medicine, Guangzhou, China; ^3^ Department of Neurology, The Second Affiliated Hospital of Guangzhou University of Chinese Medicine, Guangzhou, China; ^4^ Guangdong Provincial Key Laboratory of Research on Emergency in TCM, The Second Affiliated Hospital of Guangzhou University of Chinese Medicine, Guangzhou, China

**Keywords:** stroke-associated pneumonia, NLR, SII, PLR, SIRI, prediction value, intracerebral hemorrhage

## Abstract

**Background:**

Inflammatory mechanisms play important roles in intracerebral hemorrhage (ICH) and have been linked to the development of stroke-associated pneumonia (SAP). The neutrophil-to-lymphocyte ratio (NLR), systemic immune-inflammation index (SII), platelet-to-lymphocyte ratio (PLR) and systemic inflammation response index (SIRI) are inflammatory indexes that influence systemic inflammatory responses after stroke. In this study, we aimed to compare the predictive value of the NLR, SII, SIRI and PLR for SAP in patients with ICH to determine their application potential in the early identification of the severity of pneumonia.

**Methods:**

Patients with ICH in four hospitals were prospectively enrolled. SAP was defined according to the modified Centers for Disease Control and Prevention criteria. Data on the NLR, SII, SIRI and PLR were collected at admission, and the correlation between these factors and the clinical pulmonary infection score (CPIS) was assessed through Spearman’s analysis.

**Results:**

A total of 320 patients were enrolled in this study, among whom 126 (39.4%) developed SAP. The results of the receiver operating characteristic (ROC) analysis revealed that the NLR had the best predictive value for SAP (AUC: 0.748, 95% CI: 0.695-0.801), and this outcome remained significant after adjusting for other confounders in multivariable analysis (RR=1.090, 95% CI: 1.029-1.155). Among the four indexes, Spearman’s analysis showed that the NLR was the most highly correlated with the CPIS (r=0.537, 95% CI: 0.395-0.654). The NLR could effectively predict ICU admission (AUC: 0.732, 95% CI: 0.671-0.786), and this finding remained significant in the multivariable analysis (RR=1.049, 95% CI: 1.009-1.089, P=0.036). Nomograms were created to predict the probability of SAP occurrence and ICU admission. Furthermore, the NLR could predict a good outcome at discharge (AUC: 0.761, 95% CI: 0.707-0.8147).

**Conclusions:**

Among the four indexes, the NLR was the best predictor for SAP occurrence and a poor outcome at discharge in ICH patients. It can therefore be used for the early identification of severe SAP and to predict ICU admission.

## Introduction

Stroke-associated pneumonia (SAP) occurs in approximately 14%-27.8% of patients, and the incidence of pneumonia is relatively higher in patients with intracerebral hemorrhage (ICH) than in those with ischemic stroke (1, 2). Despite the high prevalence and clinical importance of pneumonia after ICH, few studies have investigated biomarkers for predicting SAP occurrence in patients with ICH, and studies on the prediction of severe SAP remain scarce.

Systemic inflammatory responses contribute to secondary injury in ICH and are associated with SAP. Brain damage caused by ICH initiates an inflammatory immune response that is altered through the sympathetic pathway and hypothalamus-pituitary-adrenal axis, and the continuous inflammatory reaction decreases systemic immunity activity and suppresses systemic cellular immune responses, eventually increasing the probability of SAP. Therefore, the estimation of inflammatory status helps to predict the occurrence of pneumonia in ICH patients. The neutrophil-to-lymphocyte ratio (NLR) is considered an important parameter for assessing systemic inflammatory status and infection risk and has the ability to predict community-acquired pneumonia (3, 4), infectious pathologies (5), and outcomes in patients with ICH stroke (6). The systemic immune-inflammation index (SII) is a novel biomarker of malignancy and inflammatory diseases. It has been used to predict poor outcomes following ICH and clinical outcomes of delayed cerebral vasospasm in patients with aneurysmal SAH (7, 8). The systemic inflammation response index (SIRI) indicates the balance between the inflammatory response and immune status (9) and has good predictive power for SAP in acute ischemic stroke patients (10). There is evidence that the SIRI can predict aneurismal subarachnoid hemorrhage and tumors (11). The platelet-to-lymphocyte ratio (PLR) is a marker based on platelet aggregation and systemic inflammation (12, 13). It can be employed to assess platelet activation induced by inflammatory-coagulation reactions, severe coagulation disorders and systemic inflammatory reactions. A previous study reported that the PLR may be an independent risk factor for SAP in stroke patients (14).

The objective of this study was to evaluate the predictive ability of NLR, SII, SIRI and PLR values at admission for SAP in patients with ICH and to investigate the application potential of these markers in the early identification of severe pneumonia.

## Methods

### Patients and participants

This was a multicenter prospective observational study conducted at four hospitals in Guangzhou, China (Guangdong Provincial Hospital of Chinese Medicine, Ersha Island Hospital, Fangcun Hospital and Higher Education Mega Center Hospital). Consecutive patients admitted from 15 May 2019 to 15 May 2022 were enrolled in the study. The protocol of this study was approved by the ethics committee of Guangdong Provincial Hospital of Chinese Medicine (No. ZE2019-247-01). In addition, the study is reported in line with the STROBE guidelines and was conducted in accordance with the Declaration of Helsinki.

Patients were included if they met the following criteria: aged ≥18 years old; clinically diagnosed with ICH by computed tomography; and admitted to the hospital within 48 hours of symptom onset. Patients were excluded if they met the following conditions: died within 24 hours after admission; were diagnosed with pneumonia before admission; had subarachnoid hemorrhage; had hemorrhage transformation after ischemic stroke; had a history of malignant tumor, hematologic disease, severe hepatic or renal diseases; used immunosuppressants; had active infection within the 2 weeks before admission (15); or did not provide consent to participate in the study.

### Clinical assessment

The following data were collected from the patients: demographic characteristics, anthropometric characteristics, medical history data, clinical features, and imaging information, including the Glasgow Coma Scale (GCS, with a lower score indicating a worse level of consciousness) score, the National Institute of Health Stroke Scale (NIHSS, with a higher score indicating a worse level of neurological function) score, the Clinical Pulmonary Infection Score (CPIS, with a higher score indicating more serious pneumonia), the percentage of intensive care unit (ICU) admissions, hypertension, diabetes, hyperlipidemia, and chronic obstructive pulmonary disease histories, current smoking status, and drinking status. Laboratory examinations, including routine blood tests, were performed within 24 hours of admission. We calculated the NLR, PLR, SII and SIRI according to the following equations: NLR = neutrophil count/lymphocyte count; PLR = platelet count/lymphocyte count; SII = (neutrophil count × platelet count)/lymphocyte count; and SIRI = (neutrophil count × monocyte count)/lymphocyte count.

After enrollment, ICH patients were evaluated daily for SAP occurrence. Physical examinations, close observations of respiratory symptoms and laboratory examinations were performed daily. A chest X-ray or computed tomography was performed on admission and within 6 hours of a new or worsening respiratory symptom. SAP was defined as the development of lower respiratory tract infections in nonventilated patients during the first 7 days after ICH onset according to the modified Centers for Disease Control and Prevention criteria (16). Two experienced physicians who were blinded to the clinical and laboratory results of the patients independently diagnosed SAP. Any discrepancies were resolved through discussion with a third physician. In-hospital mortality and the modified Rankin scale (mRS, with a higher score indicating a worse level of functional recovery) were recorded at discharge. A good outcome was defined as an mRS score of 0-2, and a poor outcome was defined as an mRS score of 3-6. A trained neurologist who was blinded to all clinical data evaluated the outcomes of all patients.

### Statistical analysis

Continuous variables are presented as the means ± standard deviations (SDs) or medians (interquartile ranges, IQRs) and were compared using t tests or Wilcoxon rank-sum tests, when appropriate. Categorical variables are expressed as frequencies and percentages and were compared using χ2 or Fisher exact tests, when appropriate. Age, sex, and variables that differed significantly in univariate analysis were included in multivariable logistic regression models to identify potential risk factors. Two-sided P values < 0.05 were considered statistically significant. Predictive ability was determined based on the area under the receiver operating characteristic curve (AUC). The best cutoff point was defined as that which maximized the Youden index. The correlations between variables and the CPIS were determined using Spearman’s analysis. Nomograms were developed to predict the probability of SAP occurrence and ICU admission based on the results of the multivariable logistic regression. Receiver operating characteristic (ROC) analyses and calibration curves were used to evaluate the predictive ability of the nomograms. Decision curve analyses (DCAs) were applied to determine the suitability of the nomogram for clinical application. Statistical analyses were performed using IBM SPSS 26.0, GraphPad Prism 9.0 and R version 4.2.0.

## Result

**Figure 1:**
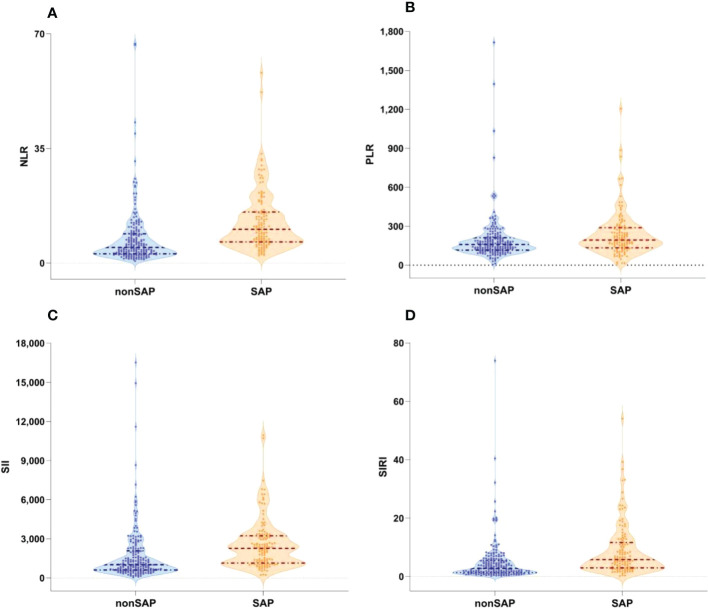
Boxplots of the NLR, PLR SII and SIRI showing the distribution in the non-SAP group (n=194) and SAP group (n=126). **(A)** The NLR of the SAP group was higher than that of the non-SAP group (<0.001). **(B)** The PLR of the SAP group was higher than that of the non-SAP group. (p=0.001). **(C)** The SII of the SAP group was higher than that of the non-SAP group. (<0.001). **(D)** The SIRI of the SAP group was higher than that of the non-SAP group(<0.001). NLR, neutrophil-to-lymphocyte ratio; SII, systemic immune-inflammation index; PLR, platelet-to-lymphocyte ratio; SIRI, systemic inflammation response index; SAP, stroke-associated pneumonia; non-SAP, non-stroke-associated pneumonia.

A total of 320 patients with ICH were included in the study; the mean age was 62.5 (51.3-73) years, and 213 (66.6%) patients were male. The initial GCS score and NIHSS score were 13 (7–15) and 11 (3–35), respectively. Among the total sample, 126 (39.4%) patients developed SAP. The baseline characteristics of the ICH patients are shown in **Table 1**. Patients with SAP showed significantly lower admission GCS scores (P < 0.001), higher NIHSS scores (P < 0.001), larger hematoma volumes (P < 0.001), and higher rates of gastric tube placement (P < 0.001) and neurosurgery (P < 0.001). A lower GCS score and higher NIHSS score indicated worse neurological function and a lower level of consciousness, meaning that the patient was suffering from severe ICH and more likely to be immunosuppressed. Patients in the SAP group showed significantly higher NLR (P < 0.001), PLR (P =0.001), SII (P < 0.001), and SIRI (P < 0.001) values, as shown in **Table 1** and **Figure 1**.

**Table 1:** Baseline characteristics of the 320 patients with ICH.

	Total (n=320)	SAP development			ICU admission		
		SAP (n=126)	Non-SAP(n=194)	P value	ICU (n=112)	Non-ICU (n=208)	P value
Demographics							
Age, years, median (IQR)	62.5 (51.3-73.0)	65.5 (52-78)	62 (50-71.25)	0.103	63.5 (51.0-74.0)	62.0 (52.0-72.0)	0.781
Sex, male, n (%)	213 (66.6%)	87 (69%)	126 (64.9%)	0.448	79 (70.5%)	134 (64.4%)	0.269
Clinical features							
GCS, median (IQR)	13 (7-15)	9 (6-12)	15 (11-15)	**<0.001**	9 (6-12)	15 (10-15)	**<0.001**
NIHSS, median (IQR)	11 (3-35)	19 (11-35)	5 (2-14)	**<0.001**	20 (12.3-35)	5 (2-14.8)	**<0.001**
Hematoma volume, ml, mean±SD	25.7 ± 20.8	32.4 ± 28.5	20.0 ± 17.6	**<0.001**	36.4 ± 29.4	19.9 ± 26.8	**<0.001**
NLR, median (IQR)	6.78 (3.68-12.27)	10.30 (6.46-15.60)	4.79 (2.82-8.95)	**<0.001**	10.2 (6.5-15.4)	4.97 (2.88-9.72)	**<0.001**
SII, median (IQR)	1349.50 (755.28-2665.64)	2271.18 (1140.25-3232.95)	1018.04 (608.02-2075.39)	**<0.001**	2271.18(1167.97-3275.65)	1033.87 (609.60-2254.91)	**<0.001**
PLR, median (IQR)	169.98 (122.03-246.61)	193.85 (133.87-288.69)	159.38 (115.96-211.16)	**0.001**	188.00 (131.45-289.16)	163.49 (119.42-217.14)	**0.008**
SIRI, median (IQR)	3.93 (1.82-7.45)	5.82 (2.96-11.67)	2.82 (1.36-5.56)	**<0.001**	6.68(2.92-11.13)	3.02 (1.53-6.00)	**<0.001**
Medical history							
Smoking, n (%)	71 (22.2%)	29 (23.0%)	42 (21.6%)	0.774	28 (25.0%)	43 (20.7%)	0.374
Drinking, n (%)	40 (12.5%)	14 (11.1%)	26 (13.4%)	0.545	13 (11.6%)	27 (13%)	0.723
Hypertension, n (%)	228 (71.3%)	87 (69%)	141 (72.7%)	0.483	76 (67.9%)	152 (73.1%)	0.325
Diabetes, n (%)	43 (13.4%)	15 (11.9%)	28 (14.4%)	0.517	16 (14.3%)	27 (13.0%)	0.744
Hyperlipidemia, n (%)	24 (7.5%)	6 (4.8)	18 (9.3%)	0.134	5 (4.5%)	19 (9.1%)	0.130
COPD, n (%)	11 (3.4%)	6 (4.8%)	5 (2.6%)	0.352	4 (3.6%)	7(3.4%)	0.923
Treatment							
Nasogastric tube, n (%)	183 (57.1%)	106(84.1%)	77(39.7%)	**<0.001**	97 (86.6%)	86 (41.3%)	**<0.001**
Neurosurgery, n (%)	96 (30.0%)	62(49.2%)	34(17.5%)	**<0.001**	60 (53.6%)	36 (11.3%)	**<0.001**

ICH, intracerebral hemorrhage; IQR, interquartile range; RR, relative risk; CI, confidence interval; GCS, Glasgow Coma Scale; NIHSS, National Institute of Health Stroke Scale; NLR, neutrophil-to-lymphocyte ratio; SII, systemic immune-inflammation index; PLR, platelet-to-lymphocyte ratio; SIRI, systemic inflammation response index; COPD, chronic obstructive pulmonary disease. The P values < 0.05 are written in bold text.

**Figure 2:**
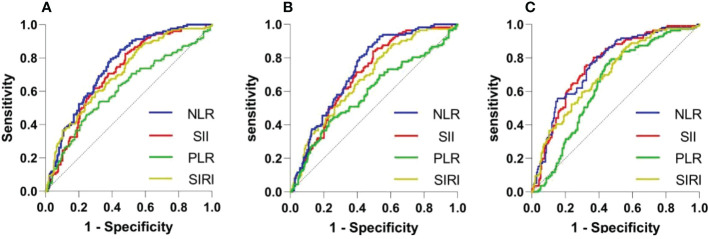
ROC curves of the NLR, PLR SII and SIRI for predicting SAP occurrence **(A)**, ICU admission **(B)** and good outcomes at discharge **(C)**. ROC, receiver operating characteristic; NLR, neutrophil-to-lymphocyte ratio; SII, systemic immune-inflammation index; PLR, platelet-to-lymphocyte ratio; SIRI, systemic inflammation response index; SAP, stroke-associated pneumonia; ICU, intensive care unit.

ROC analysis was conducted to determine the ability of the biomarkers to predict SAP (**Figure 2**). Based on the AUC values, the NLR (AUC: 0.748, 95% CI: 0.695-0.801), SII (AUC: 0.707, 95% CI: 0.650-0.763), and SIRI (AUC: 0.707, 95% CI: 0.650-0.764) showed higher predictive ability for SAP in patients with ICH than the PLR (AUC: 0.612, 95% CI: 0.548-0.677). Details of the optimal cutoff, specificity and sensitivity rates are shown in **Table 2**. In the multivariable logistic regression analysis, the admission NLR value (adjusted relative risk [RR]=1.090, 95% confidence interval [CI]: 1.029-1.155, P=0.003) remained significant after adjusting for other confounders, including age and sex. Baseline variables that showed significant variations were included in the logistic regression analyses (**Table 3**) . High SII (unadjusted RR=1.000, 95% CI: 1.000-1.001, P=0.001) and SIRI (unadjusted RR=1.076, 95% CI: 1.037-1.116, P<0.001) values were found to be risk factors in the unadjusted model.

**Table 2:** AUC in predicting SAP occurrence, ICU admission and good outcomes at discharge.

	AUC (95% CI)	Cutoff point	Sensitivity	Specificity	PPV	NPV
AUC in predicting SAP occurrence						
NLR	0.748 (0.695-0.801)	6.06	0.794	0.608	0.568	0.819
SII	0.707 (0.650-0.763)	1039	0.825	0.521	0.528	0.821
PLR	0.612 (0.548-0.677)	187.5	0.540	0.675	0.519	0.693
SIRI	0.707 (0.650-0.764)	2.291	0.500	0.438	0.865	0.833
AUC in predicting ICU admission						
NLR	0.732 (0.671-0.786)	5.50	0.866	0.534	0.495	0.874
SII	0.701 (0.644-0.758)	1039	0.839	0.505	0.475	0.854
PLR	0.590 (0.522-0.657)	222.9	0.420	0.769	0.495	0.714
SIRI	0.680 (0.620-0.734)	2.802	0.795	0.481	0.447	0.807
AUC in predicting good outcomes at discharge						
NLR	0.761 (0.707-0.814)	7.19	0.811	0.612	0.523	0.858
SII	0.758 (0.705-0.812)	1384	0.802	0.632	0.532	0.792
PLR	0.642 (0.581-0.703)	185.1	0.784	0.536	0.473	0.834
SIRI	0.722 (0.665-0.779)	2.676	0.595	0.751	0.217	0.394

AUC, area under receiver operating characteristic; NLR, neutrophil-to-lymphocyte ratio; SII, systemic immune-inflammation index; PLR, platelet-to-lymphocyte ratio; SIRI, systemic inflammation response index; SAP, stroke-associated pneumonia; ICU, intensive care unit.

**Table 3:** Logistic regression models for SAP occurrence and ICU admission.

Covariates	Univariate		Multivariable		Multivariable (Reduced)	
	Crude RR (95% CI)	P value	Adjusted RR (95% CI)	P value	Adjusted RR (95% CI)	P value
SAP occurrence						
Sex, male	1.204(0.746-1.944)	0.448	1.465(0.788-2.725)	0.228		
Age, years	1.013(0.998-1.028)	0.088	1.023(1.003-1.043)	**0.027**	1.020(1.001-1.040)	**0.036**
Admission GCS score	0.817(0.771-0.867)	**<0.001**	0.984(0.840-1.152)	0.838		
Admission NIHSS score	1.068(1.049-1.088)	**<0.001**	1.039(0.987-1.093)	0.143	1.042(1.015-1.069)	**0.002**
Hematoma volume	1.018(1.010-1.027)	**<0.001**	0.989(0.978-1.000)	0.061	0.990(0.978-1.001)	0.071
Nasogastric tube	8.053(4.611-14.066)	**<0.001**	2.984(1.481-6.012)	**0.002**	2.901(1.446-5.820)	**0.003**
Neurosurgery	4.631(2.781-7.711)	**<0.001**	3.661(1.994-6.722)	**<0.001**	3.701(2.018-6.787)	**<0.001**
NLR	1.074(1.040-1.109)	**<0.001**	1.078(1.005-1.156)	**0.035**	1.090(1.029-1.155)	**0.003**
PLR	1.001(1.000-1.003)	0.057	0.997(0.994-1.001)	0.115	0.997(0.994-1.000)	**0.026**
SII	1.000(1.000-1.001)	**0.001**	1.000(1.000-1.001)	0.671		
SIRI	1.076(1.037-1.116)	**<0.001**	1.020(0.971-1.071)	0.434		
ICU admission						
Sex, male	1.322 (0.805-2.170)	0.270	1.575 (0.831-2.986)	0.163		
Age, years	1.003 (0.988-1.018)	0.722	1.012 (0.991-1.032)	0.261		
Admission GCS score	0.809 (0.761-0.859)	**<0.001**	0.982 (0.838-1.151)	0.820		
Admission NIHSS score	1.072 (1.052-1.092)	**<0.001**	1.039 (0.987-1.094)	0.141	1.040 (1.017-1.064)	**0.001**
Hematoma volume	1.020 (1.011-1.029)	**<0.001**	0.993 (0.982-1.004)	0.215		
Nasogastric tube	9.175 (4.996-16.850)	**<0.001**	3.388 (1.605-7.153)	**0.001**	3.913 (1.917-7.987)	**<0.001**
Neurosurgery	5.621 (3.348-9.436)	**<0.001**	4.069 (2.233-7.413)	**<0.001**	3.552 (2.010-6.275)	**<0.001**
NLR	1.056 (1.026-1.086)	**<0.001**	1.051 (1.011-1.091)	**0.027**	1.049 (1.009-1.089)	**0.036**
PLR	1.001 (1.000-1.002)	0.156	0.997 (0.994-1.000)	0.076		
SII	1.000 (1.000-1.001)	**0.001**	1.000(1.000-1.001)	0.444		
SIRI	1.053 (1.020-1.087)	**0.002**	0.997 (0.953-1.043)	0.893		

RR, relative risk; CI, confidence interval; GCS, Glasgow Coma Scale; NIHSS, National Institute of Health Stroke Scale; NLR, neutrophil-to-lymphocyte ratio; SII, systemic immune-inflammation index; PLR, platelet-to-lymphocyte ratio; SIRI, systemic inflammation response index; SAP, stroke-associated pneumonia; ICU, intensive care unit. The P values < 0.05 are written in bold text.

**Figure 3:**
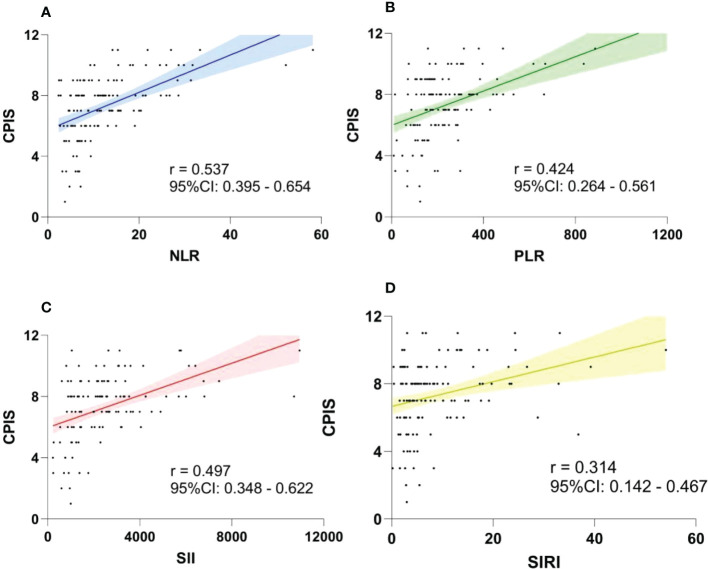
Association between the CPIS and NLR **(A)**, PLR **(B)**, SII **(C)** and SIRI **(D)** using Spearman’s analysis. The NLR had the highest correlation coefficient. CPIS, clinical pulmonary infection score; CI, confidence interval; NLR, neutrophil-to-lymphocyte ratio; SII, systemic immune-inflammation index; PLR, platelet-to-lymphocyte ratio; SIRI, systemic inflammation response index.

The CPIS is a scoring system that integrates clinical, imaging, and microbiological criteria for assessing the severity of infection. The relationship between the CPIS and four indexes was evaluated through Spearman’s analysis (**Figure 3**). Among the four indexes, the NLR (correlation coefficient r=0.537, 95% CI: 0.395-0.654) showed the highest correlation with the CPIS, indicating that the NLR was significantly correlated with the CPIS.

A total of 112 patients (35.0%) were admitted to the ICU, among whom 110 (34.4%) were diagnosed with SAP (**Table 1**). Patients admitted to the ICU also showed significantly lower admission GCS (P < 0.001) and higher NIHSS (P < 0.001) scores because they had a greater need for intubation and mechanical ventilation. ROC analysis revealed that the NLR (AUC: 0.732, 95% CI: 0.671-0.786) and SII (AUC: 0.701, 95% CI: 0.644-0.758) had good predictive power for ICU admission and exceeded that of the SIRI (AUC: 0.680, 95% CI: 0.620-0.734) and PLR (AUC: 0.590, 95% CI: 0.522-0.657). In the multivariable logistic regression analysis, the admission NLR (RR=1.049, 95% CI: 1.009-1.089, P=0.036) value remained significant after adjusting for other confounders (**Table 3**).

**Figure 4:**
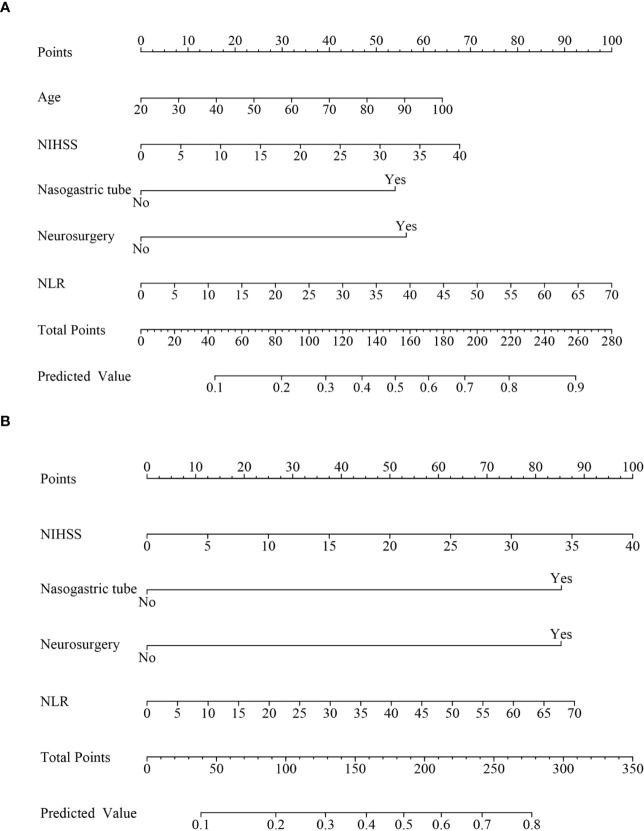
Nomogram to estimate the risk of SAP in patients with ICH. **(A)** Nomogram model to predict SAP occurrence. **(B)** Nomogram model to predict ICU admission. ICH, intracerebral hemorrhage; SAP, stroke-associated pneumonia; ICU, intensive care unit.

Nomograms were developed to predict the probability of SAP occurrence and ICU admission (**Figure 4**). Points were attributed to five individual variables in the nomogram of SAP occurrence and four individual variables in the nomogram of ICU admission. The nomograms allowed direct reading of the probability after summing the points for each single predictor. The AUC was 0.815 (95% CI =0.769-0.860) for SAP occurrence and 0.828 (95% CI =0.785-0.872) for ICU admission (**Supplemental Figure 1**), showing that the predictive accuracy of the nomograms was acceptable. The calibration curves showed a favorable consistency between the predicted and actual probabilities (**Supplemental Figure 2**). As the DCA results indicated, the models had a net benefit when the threshold probabilities were < 71% in the nomogram for SAP occurrence and <65% in the nomogram for ICU admission (**Supplemental Figure 3**).

Clinical outcomes included in-hospital mortality and mRS score at discharge (**Figure 2**). In-hospital mortality was significantly higher in patients with pneumonia (24 [19.0%]) than in those without pneumonia (20 [10.3%]) (P=0.027). Similarly, patients with SAP showed higher rates of poor outcomes (107 [84.9%]) than those without SAP (102 [52.6%]) (<0.001). ROC analysis showed that the NLR (AUC: 0.761, 95% CI: 0.707-0.8147), SII (AUC: 0.758, 95% CI: 0.705-0.812), and SIRI (AUC: 0.722, 95% CI: 0.665-0.779) predicted poor outcomes at discharge, as shown in **Figure 2** and **Table 2**.

## Discussion

SAP is a common and threatening complication after stroke, especially in patients with ICH. However, there are few clinical studies on SAP in patients with ICH compared with those in patients with acute ischemic stroke. To our knowledge, no study has reported markers for the early identification of SAP severity in ICH patients. In this prospective study, for the first time, we compared the predictive value of the NLR, SII, SIRI and PLR for SAP and investigated the relationship between these four inflammatory indexes and SAP severity, which was assessed by the CPIS. Our results showed that SAP occurred in 39.4% of the ICH patients. Moreover, the NLR, SII and SIRI showed high prediction accuracy for SAP and poor neurological outcomes at discharge. The NLR was strongly related to SAP severity. Our findings are expected to improve the early prediction and identification of SAP and its severity in patients with ICH.

The majority of SAP cases occurred within the first 7 days after stroke onset, with a peak incidence on the third day (17). This suggests that early identification of SAP in patients with ICH is crucial for effective treatment. The inflammatory response increases rapidly after stroke onset and is strongly linked to the occurrence of SAP (18). Inflammatory biomarkers such as the NLR, SII, SIRI and PLR can reflect the state of systemic inflammation and the immune response (10). Neurological injury induced by the inflammatory response and the release of cytokines from immune cells results in the production of anti-inflammatory signals and inhibits cytokine production, ultimately inhibiting infection and preventing disease progression (19). However, a sustained inflammatory response eventually exhausts the immune system (20), which decreases systemic immunity activity, suppresses systemic cellular immune responses (21), and induces a rapid decrease in peripheral blood lymphocyte subpopulations, which is known as stroke-induced immunodepression syndrome (SIDS). This increases a patient’s susceptibility to SAP.

In this study, we analyzed the predictive ability of four indexes for SAP and severity. The results showed that the NLR, SII and SIRI showed good predictive ability for SAP with AUCs of more than 0.700, and the NLR was an independent risk factor in the adjusted multivariable logistic regression analysis, indicating that the NLR had the best predictive power among the four indexes. Therefore, NLR, SII and SIRI values, especially NLR values, should be calculated at admission for patients with ICH to identify patients who require stronger respiratory support and intervention for respiratory infections to ensure a good prognosis (22–25). In this study, we found that patients with SAP had significantly lower admission GCS scores, higher admission NIHSS scores, and larger hematoma volumes, indicating that they had severe brain damage and independent risk factors for SAP. A lower admission GCS score and higher admission NIHSS score indicated more severe brain damage and neurological disturbances, causing serious immunosuppression and leading to the development of SAP. Acute brain injury patients with a GCS score below eight were at higher risk of respiratory depression, it was strongly recommended that these patients be admitted to the ICU for monitoring and receive mechanical ventilation. It has been reported that gastric tube placement can increase susceptibility to SAP by elevating the risk of gastroesophageal reflux and aspiration (26). Patients who underwent neurosurgery were more likely to develop aspiration and SAP because the cough reflex and tracheal ciliary protective movement were inhibited by the neuromuscular block produced by general anesthesia (27, 28).

The focus of this investigation was to explore the correlation between four indexes and SAP severity. The CPIS comprises clinical features, laboratory tests, imaging and microbiological criteria for assessing SAP infection, with a maximum score of twelve. A high CPIS score indicates severe infection that requires aggressive anti-infection treatment (13, 29, 30). We analyzed the correlation coefficients of the NLR, PLR, SII and SIRI with the CPIS and found that the NLR had the strongest correlation. In addition, the percentage of ICU admission partly reflected SAP severity, as supported by data from 110 of the 112 ICU patients who were diagnosed with SAP. ICH patients with SAP were more likely to develop critical symptoms such as respiratory and circulatory failure, requiring mechanical ventilation and monitoring. ROC analysis revealed that the NLR exhibited the best prediction of ICU admission, and adjusted multivariable logistic regression showed that the NLR was an independent risk factor. Our findings suggested that the NLR value at admission improved the early identification of severe pneumonia and ICU admission. Thus, patients with a high NLR value should receive airway management and early anti-infection intervention to avoid deterioration.

There are several limitations in this study. First, data on 90-day mRS scores were lacking; hence, long-term prediction could not be conducted. Previous research has demonstrated that the NLR can predict long-term outcomes of ICH, but our study only shows the predictive power of the SII and SIRI for mRS scores at discharge. The small sample size of this study may also introduce confounding bias. In addition, we only recorded inflammatory indexes within 24 hours after admission; however, the inflammatory response may evolve within a few days of ICH onset. Thus, future studies are needed to explore the changes and predictive value of inflammatory factors at different time points after onset.

## Conclusion

The results of this study indicate that the NLR, SII and SIRI can predict the occurrence of SAP and poor outcomes at discharge in ICH patients. Among these variables, the NLR has the best prediction performance. The NLR may also facilitate early identification of severe SAP and predict ICU admission.

## Data availability statement

The original contributions presented in the study are included in the article/**Supplementary Material**. Further inquiries can be directed to the corresponding authors.

## Ethics statement

The studies involving human participants were reviewed and approved by the Ethics Committee of the Second Affiliated Hospital of Guangzhou University of Chinese Medicine approved this study (No. ZE2019-247-01). The patients/participants provided their written informed consent to participate in this study.

## Author contributions

The authors’roles are as follows. Conception and design: L-XW, H-JL, R-HW. Methodology: H-JL, FY, R-HW. Acquisition and interpretation of the data: R-HW, H-PL, Z-HM, A-LL, Z-PJ, W-XW, Y-FC, J-WG. Analysis of data: R-HW, W-XW, Z-PJ. Drafting the manuscript: R-HW, H-JL, Z-PD, S-BW. Revising the manuscript: L-XW, FY, R-HW, YH, A-LL, Z-HM, H-JL. Obtained funding: L-XW, H-JL, W-XW. Supervision: L-XW, YH. Final approval: All authors provided final approval of the manuscript.
