# Supplementary material for: The clinical value of neutrophil-to-lymphocyte ratio (NLR), systemic immune-inflammation index (SII), platelet-to-lymphocyte ratio (PLR) and systemic inflammation response index (SIRI) for predicting the occurrence and severity of pneumonia in patients with intracerebral hemorrhage
Source: Front Immunol. 2023 Feb 13;14:1115031. doi: 10.3389/fimmu.2023.1115031 (PMC9969881; doi:10.3389/fimmu.2023.1115031)
Supplement: Supplementary file 1 [file DataSheet_1.docx]

**Supplemental Materials**

**Supplemental Figure 1.** Calibration curves of the nomogram model.

**Supplemental Figure 2.** ROC curve of the nomogram.

**Supplemental Figure 3.** DCA of the nomogram.

**Supplemental Figure 1.** Calibration curves of the nomogram model. **(A)** Calibration curve for the SAP occurrence model. **(B)** Calibration curve for the ICU admission model. SAP, stroke-associated pneumonia; ICU, intensive care unit.

**Supplemental Figure 2.** ROC curve of the nomogram. **(A)** ROC curve for the SAP occurrence model. **(B)** ROC curve for the ICU admission model. ROC, receiver operating characteristic; SAP, stroke-associated pneumonia; ICU, intensive care unit.

**Supplemental Figure 3.** DCA of the nomogram. **(A)** DCA of the SAP occurrence model. **(B)** DCA of the ICU admission model. DCA, decision curve analysis; SAP, stroke-associated pneumonia; ICU, intensive care unit.
